# Incidental Finding of a Periampullary Neuroendocrine Tumor: A Case Report

**DOI:** 10.7759/cureus.35198

**Published:** 2023-02-19

**Authors:** Anjala Nizam, Nadia M Saleem, Tiba A Albakri, Amir Saber, Rabia Farhan

**Affiliations:** 1 Department of Medicine and Surgery, Dubai Academic Health Corporation, Dubai, ARE; 2 Department of General Surgery, Rashid Hospital, Dubai, ARE; 3 Department of Pathology and Genetics, Dubai Hospital, Dubai, ARE

**Keywords:** whipple procedure, periampullary tumor, carcinoid tumor, ampullary tumor, incidental gastrointestinal neuroendocrine tumor, gastrointestinal neuroendocrine tumor

## Abstract

A 63-year-old male with multiple co-morbidities presented with a diabetic foot infection which was treated surgically. During admission to the hospital, he developed melena and underwent an endoscopic assessment which revealed an incidental finding of an ampullary mass. The histological analysis of the biopsy revealed ampullary carcinoma with mixed intestinal-type and pancreatobiliary-type features. A magnetic resonance imaging (MRI) of the liver with contrast presented the tumor as an ill-defined small soft tissue lesion measuring 8 x 9 mm in the ampullary region, with multiple lymph nodes in the periportal, peripancreatic, and para-aortic regions. There was no evidence of biliary obstruction. The patient underwent a Whipple procedure with no complications. The final histology report of the specimens taken stated that the tumor is predominantly in the duodenum and focally in the ampulla, and is a well-differentiated neuroendocrine tumor confirmed to be submucosal. The histopathologic and radiologic workup determined the pathological stage classification to be pT3N1, Mx G1.

## Introduction

Gastroenteric neuroendocrine tumors (NETs), previously called carcinoids, are rare [1,2]. They vary in their clinical presentation, histological morphology, biological activity, and prognosis. Those tumors located at the ampulla of Vater represent a minority of the gastroenteric NETs but they tend to be more aggressive [3]. They may present with jaundice, anemia, gastrointestinal bleeding, gastric outlet obstruction, or incidental discovery [4]. The treatment of choice usually involves the complete surgical resection of the tumor, but endoscopic papillectomy may be opted for in low-grade cases with no metastasis or invasion of the biliary ducts [5].

## Case presentation

**Figure 1:**
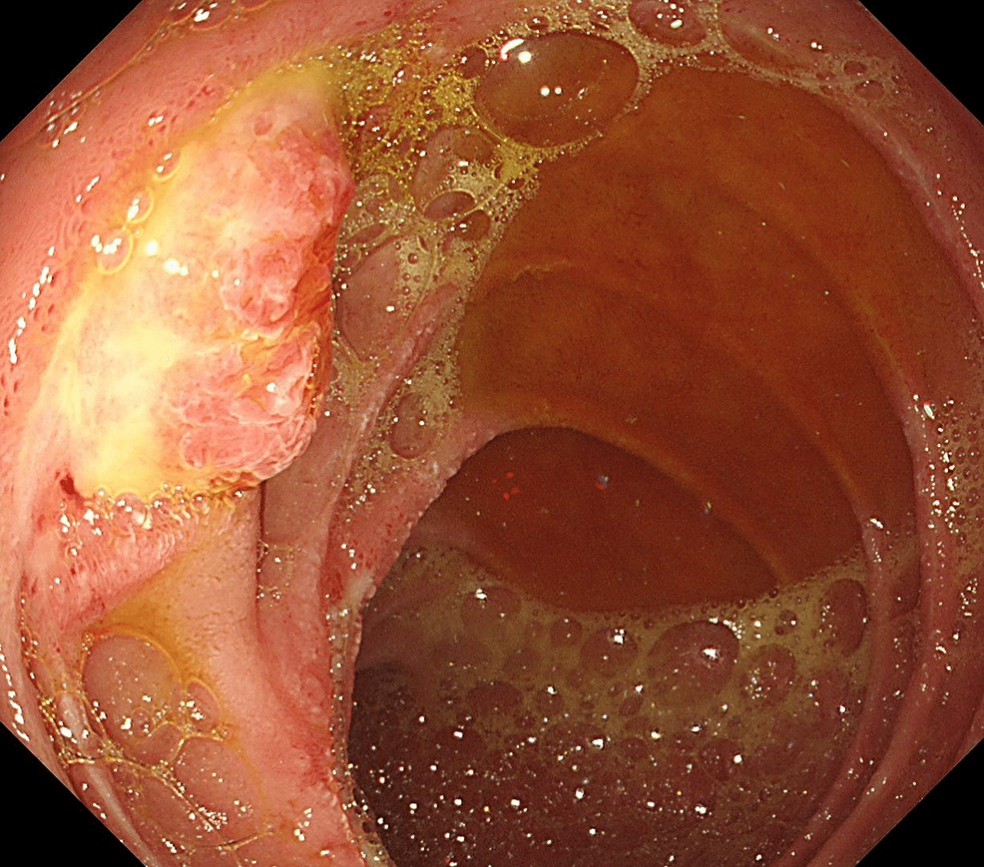
Endoscopic imaging showing an ampullary mass

A 63-year-old male patient was admitted as a case of a left diabetic foot infection and underwent debridement and Lisfrank amputation, followed by vacuum-assisted closure of the wound. His past medical history was significant for hypertension, type 2 diabetes mellitus, and dyslipidemia, whereas his surgical history was significant for umbilical hernia repair, cholecystectomy, and right nephrectomy. On postoperative day six, the patient passed black tarry stools and became tachypneic and tachycardic. His hemoglobin level dropped from 8.7 g/dL to 3.8 g/dL and he was given six units of blood transfusion, after which he underwent a gastroscopy to determine the source of the gastrointestinal bleed. The endoscopic assessment revealed multiple duodenal ulcers (Forrest classification: llA and lll) and a suspected ampullary mass from which a biopsy was taken (Figure 1).

**Figure 2:**
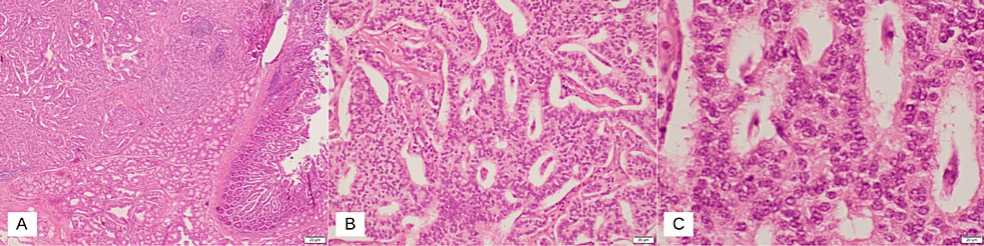
Histopathology slides of the duodenal mucosa showing the neuroendocrine tumor: A. Low-power view (10x). B. High-power view (40x). C. High-power view (40x)

**Figure 3:**
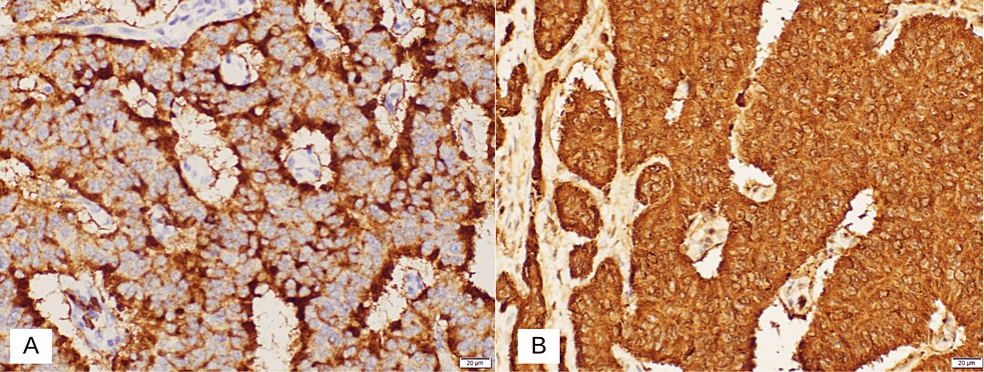
Histopathology slides of duodenal mucosa showing tumor cell staining with: A. chromogranin immunostaining. B. CD56 immunomarker

**Figure 4:**
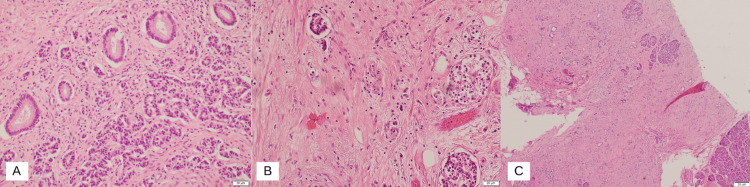
Histopathology sides of specimens: A. Low-power view (10x) showing ampullary tissue showing nests and clusters of neuroendocrine tumor cells. B. Tumor cells within the lymphovascular spaces. C. Tumor cells infiltrating pancreatic tissue

Contrast-enhanced computed tomography (CT) scan of the abdomen and pelvis with coronal reformats in the early arterial phase showed an ill-defined hypodense prominence of the ampullary region suspicious for an ampullary tumor, with no upstream dilatation of the common bile duct or pancreatic duct. Histological analysis with immunohistochemistry of the biopsy revealed ampullary carcinoma with mixed intestinal-type and pancreatobiliary-type features. A magnetic resonance imaging (MRI) of the liver with contrast showed an ill-defined small soft tissue lesion measuring 8 x 9 mm in the ampullary region. Multiple lymph nodes were noted in the periportal, peripancreatic, and para-aortic regions. The common bile duct (CBD) and main pancreatic duct were clearly traceable up to its confluence with no definite mass lesion and no evidence of biliary obstruction. The patient underwent the Whipple procedure with no complications. A hard mass was felt in the second part of the duodenum with multiple large (>2 cms) porta hepatis lymphadenopathy which was excised and sent for histopathological analysis. There were no signs of hepatic or peritoneal deposits of secondaries. The abdomen was closed with two drains inserted, one near the pancreatic and CBD anastomosis and the other near the gastrojejunostomy. The drains had minimal output in the postoperative period. The final histology report of the specimens taken stated that the tumor, which is predominantly in the duodenum and focally in the ampulla, is a well-differentiated neuroendocrine tumor confirmed to be submucosal. Immunohistochemical stains were performed using chromogranin, synaptophysin, CD56, CKAEA/AE3, CK20, CDX2, PAX8, S100, and Ki-67 (Figures 2-4). The results are shown in Table 1.

**Table 1:** Immunohistochemical stains

Immunohistochemical stain	Positive/Negative
Chromogranin	Positive
Synaptophysin	Positive
CD56	Positive
CKAEA/AE3	Positive
CK20	Negative
CDX2	Negative
PAX8	Negative
S100	Negative
Ki-67	Negative

The proliferation index by Ki-67 was approximately 1%. One (paraduodenal) out of the 20 regional lymph nodes was positive for metastasis. The tumor was unifocally invading the pancreatic parenchyma and peripancreatic soft tissue. There was also lymphovascular and perineural invasion. The pathological stage classification (pTNM, AJCC 8th Edition) was pT3N1, Mx G1. His postoperative recovery had been uneventful and he was discharged home in stable condition. The chromogranin A level was found to be 3180 μg postoperatively and he was started on Creon by the oncology team. During follow-up visits in the outpatient setting, the patient denied malaise, weight loss, and other complications of the Whipple procedure.

## Discussion

Neuroendocrine tumors are genetically diverse slow-growing tumors characterized by peptides that cause hormonal syndromes. The gastrointestinal (GI) tract accounts for 60% of primary tumors, followed by the tracheobronchial tree for 25%. Within the GI tract, carcinoid tumors most commonly occur in the small intestine. NETs at the ampulla of Vater are extremely rare, with studies showing an incidence of <1% to <0.05% of gastrointestinal NETs [6-8]. Annually, the prevalence of GI carcinoids is 35 per 100,000, mainly seen in patients between 50 to 70 years old, with males and females affected equally [9]. A study by Ruff et al. using the national cancer database between 2004-2016 showed that only 872 patients had ampullary NETs (as compared to 9692 and 6562 cases of duodenal and pancreatic head NETs respectively) [7]. 

Patients with ampullary carcinoids usually present with jaundice. However, other symptoms may include abdominal pain, malaise, steatorrhea, weight loss, or melena due to GI bleed. Pancreatitis was also the chief complaint in some cases [6,10]. Early stages of the disease usually result in only vague symptoms, thus diagnosis may be made following an incidental finding rather than being guided by patient symptoms [8]. Patients with suspected ampullary NETs may undergo both anatomical imaging such as CT or MRI as well as functional somatostatin receptor imaging such as 68Ga-DOTATATE positron emission tomography (PET) scan or 64Cu-DOTATATE for diagnostic as well as staging purposes [11]. Endoscopic ultrasounds, fine needle aspiration cytology, and endoscopic retrograde cholangiopancreatography (ERCP) are the most effective methods to make a diagnosis of GI NET. Due to the origin of the tumor often being deep, the diagnosis can be hard to make and biopsy results may often be negative due to the overlying normal duodenal mucosa [5]. However, endoscopic ampullectomy aids diagnosis and in some cases may even be curative [8,10]. 

Ampullary NETs that are well-differentiated have a 10-year survival rate of 71% compared to 15% in those that are poorly differentiated [8]. Chromogranin A is an important tumor marker associated with it which is also useful in tumor volume determining as well as progression and disease response, and levels > 5000 μg/L indicate poor prognosis [6,9]. If positive, 24-hour 5-hydroxyindoleacetic acid (5-HIAA) and pancreastatin (levels not affected by proton pump inhibitor use) should also be checked [9]. Ampullary NETs with increased expression of Ki-67 and proliferating cell nuclear antigen (PCNA) are more aggressive and have a higher risk of metastasis [8].

Treatment of NETs depends on the severity, which is assessed by the symptoms, the patient's general condition, and age, as well as the tumor’s grade, stage, and site. The Whipple procedure along with lymphadenectomy is the cornerstone of management for patients who are able to undergo surgery since aggressive operative extirpation provides the only chance for a cure [8,10]. Biological treatment with somatostatin analogs such as octreotide and lanreotide can be applied in symptomatic patients with slowly growing neoplasms. They may also be employed in metastatic cases, with some studies recommending prophylactic cholecystectomy in patients receiving these medications long-term as they increase the risk of biliary sludge and gallstones. Other biologic therapies include everolimus which is an oral mammalian target of rapamycin (mTOR) inhibitor and has been shown to decrease the risk of progression of the disease [12]. New tumor-targeted radiotherapy has been introduced recently with favorable results. This proposed treatment modality targets tumors with peptide receptor radionuclide therapy. It works by radiolabelling the somatostatin analogue 177-Lutetium which aggregates at the NET site to exert a cytotoxic effect [12]. Future therapy may be guided by specific tumor biology and treatment can be tailored toward individual patients [6]. For non-functional, asymptomatic, and low-volume metastatic diseases, expectant management and observation along with regular imaging are adequate [12].

## Conclusions

The unusual location of the NET in the ampullary region of our patient made it an interesting and challenging case. However, despite undergoing major surgery such as the Whipple procedure at an advanced age, the patient made an uneventful recovery. Though there have been a few studies on ampullary NETs, the information available is quite limited, hence, there exists a dire need for further research into this topic. 
